# Two Case Reports of Biliary Tract Injuries during Laparoscopic Cholecystectomy

**DOI:** 10.5402/2011/868471

**Published:** 2011-02-21

**Authors:** O. Romano, C. Romano, D. Cerbone, P. Sperlongano, L. Caserta, N. Frega, G. Cimmino, A. D'Agostino, R. Addeo

**Affiliations:** ^1^General Surgery Division, “Cardinale Ascalesi” Hospital, Via egiziaca a Forcella, 80100 Naples, Italy; ^2^Department of Anaesthesiology and Special Surgery, Second University of Naples, 80100 Naples, Italy; ^3^Gastroenterology Unit San Giovanni di Dio Hospital, Frattamaggiore, 80100 Naples, Italy; ^4^Surgery Unit, “Mauro Scarlato” Hospital, 84018 Scafati, Italy; ^5^Unit of General Surgery, San Giovanni di Dio Hospital, Frattamaggiore, 80027 Naples, Italy; ^6^San Giovanni di Dio Hospital, Frattamaggiore, 80027 Naples, Italy; ^7^Unit of Surgery, Loreto Mare Hospital, 80100 Naples, Italy; ^8^Unit of Day Hospital Oncology, San Giovanni di Dio Hospital, via Pirozzi, 53, Frattamaggiore, 80027 Naples, Italy

## Abstract

*Background and Study Aims*. Biliary tract injuries (BTI) represent the most serious and potentially life-threatening complication of cholecystectomy occurring also during laparoscopic approaches. *Patients and Methods*. We describe and discuss two different cases of BTI occurring during laparoscopic cholecystectomy (LC). *Results*. Two patients developed BTI during LC and one evidenced the complication during the LC itself and was treated during the same LC in real time. The other patient evidenced BTI only after the primary intervention and was successfully reoperated in laparotomy after 10 days from the LC. *Conclusions*. The factors that predispose to the occurrence of BTI during cholecystectomy and the cautions to be used to prevent BTI are discussed.

## 1. Introduction

Biliary tract injuries (BTI) represents the most serious and potentially life-threatening complication of cholecystectomy. Since the introduction of laparoscopic cholecystectomy (LC) in 1987 by Philippe Mouret in France [1], an increase in these iatrogenic injuries has been observed worldwide. During open cholecystectomies (OC), the prevalence of bile duct injuries has been estimated at only 0.1-0.2% (Table 1) [2–7]. The risk factors during OC include, (i) surgeon's learning curve, (ii) acute or scleroatrophic cholecystitis, (iii) misidentified anatomy, (iv) misinterpreted or incomplete cholangiography, and (v) anatomical abnormalities and excessive bleeding. 

Biliary anatomical variations are encountered in 18–39% of cases, with potentially hazardous anomalies predisposing to BTI in only 3–6%. Anomalous right hepatic ducts are considered the most dangerous type of anomaly. The injuries occurring during OC include partial or complete transaction or wide resection. The bile duct reconstruction exposes to risk of stenosis, that needs delicate surgical approaches.

The advantages of the laparoscopic procedure include minimal scarring and short postoperative recovery. However, a proportion of cases will require conversion to an open laparotomy. It is important to identify patients at higher risk of conversion preoperatively to allow appropriate patient counseling and planning of resources. Previous studies have identified parameters such as advancing age, male sex, acute cholecystitis, and others, as independent risk factors for conversion. The challenge is to reliably identify acute cholecystitis clinically, because studies have shown that there is a poor correlation between the clinical and pathologic diagnosis of acute cholecystitis. The second issue in laparoscopic cholecystectomy for acute cholecystitis is the timing of surgery. The recent literature suggests that prompt laparoscopic cholecystectomy in the acute phase does not have higher conversion rates than interval surgery. Patients with choledocholithiasis had magnetic resonance cholangiopancreatography or endoscopic retrograde cholangiopancreatography (ERCP) performed and underwent preoperative endoscopic sphincterotomy (ES) [8]. The majority of operations were performed by consultant surgeons with a minimum of 10 years experience in performing laparoscopic cholecystectomy.

The introduction of LC is associated with a significantly increased risk of BTI. In an American state-wide survey of the Connecticut which included 30,211 patients, the incidence of BTI increased from 0.04% in 1989 to 0.24% in 1991, corresponding to the introduction of LC, but then decreased to 0.11% in 1993 [9].

We described two cases of post-LC BTI that were both repaired one with laparoscopy in real time and in laparotomy after 1 month from the first intervention, respectively. In our institutions, LC indications comprehend the treatment of chronic and acute cholecystitis.

## 2. Case Reports

**Case 1**

On April 2010, a male patient of 45 years old affected by symptomatic cholelithiasis came at our observation. The patient had not jaundice or other risk factors and, therefore, was enrolled for a laparoscopic cholecystectomy. During the dissection of triangle of Calot, a partial resection of biliary common duct was made. Immediately, the BTI was evident and sheltered in laparoscopy, suturing with a resorbable spin, without biliary drainage. The postoperative outcome was good, without alterations of any laboratory or clinical parameters, and the patient was dismissed after three days. At the last followup (September 2010), the cholangiography did not show stenosis or leakage.

**Case 2**

On June 2010, a female patient of 55 years old was admitted in our Unit showing a cholecystitis with gallstones. The patient did not present jaundice, but abdominal pain, leucocytosis, and fever. Moreover, ultrasonography evidenced a parietal flogosis. LC was performed after five days; during operation, common biliary duct was misidentified, for subverted anatomy caused by inflammation. The coledochos was clipped, and the patient presented jaundice after three days after operation. The cholangiography was performed demonstrating the obstruction of the coledochos. Therefore, a reoperation was required and laparotomic Roux-en-Y hepatico-jejunostomy was performed.The patient was demitted in eleven days, with a Kehr drainage. After 40 days, the drainage was removed and last control (September 2010) evidenced the normality of haematic parameters and good clinical conditions.

## 3. Discussion

The most common cause of BTI is the failure to recognize the anatomy of the triangle of Calot. This is attributed to factors inherent to the laparoscopic approach, to inadequate training of the surgeon, and to local anatomical risk factors. Inherent risk factors of the laparoscopic approach are: (i) limitation of two-dimensional vision, (ii) absence of manual palpation of the porta and hepatis veins, (iii) use of tangential and lower approaches to the common biliary duct, and (iv) reduced visual field during a significant bleeding. The laparoscopic “learning curve” of the surgeon is the most important factor of bile duct injury [10]. But also local anatomical risk factors are important such as acute cholecystitis [11, 12], severe chronic scarring of the gallbladder [10, 13], and bleeding or excessive fat in the hepatic hilum [14]. These local risk factors seem to be present in 15% to 35% of BTI [15]. Abnormal biliary anatomy, such as a short cystic duct or a cystic duct entering into the right hepatic duct are common and also may increase the incidence of BTIs [13, 16]. 

Some authors have also underlined the importance of a right hepatic arterial anomaly running parallel to the cystic duct such as an anomalous or accessory right hepatic artery [17, 18]. Schematic representation of the common mechanisms of BTI during LC are: (i) misidentification of the cystic duct and the common hepatic duct, (ii) lateral clipping of the common hepatic duct, (iii) traumatic avulsion of the cystic duct junction, (iv) diatermic injury of common hepatic duct during dissection of the “Calot” or during the cholecystectomy, and (v) injury of anomalous right hepatic duct.

The role of intraoperative cholangiography (IOC) in the prevention of BTI remains controversial [19–21]. The greatest value of IOC is in detecting the biliary anatomy and showing the severity of BTI.

In the first described case, both the evidence of a lesion and the partial damage have allowed the reparation of the lesion during the laparoscopy in real time with a simple suture without the occurrence of complications. In the second case, lesion was evidenced after the ending of the laparoscopy with the occurrence of postoperative jaundice. The analysis of the VHS recording of the intervention has allowed the identification of the injury, and the intraoperative cholangiography confirmed the clipping of common hepatic duct.

The most frequent procedure used for biliary repair is Roux-en-Y hepatico-jejunostomy. Attention has to be given to a mucosa-to-mucosa hepato-enteric anastomosis to prevent recurrent bile duct stenosis. End-to-end choledocho-choledochostomy, usually over a T-tube, is another surgical alternative providing that a microsurgical anastomosis on healthy biliary tissue is possible; there is absence of wide bile duct excision and of any tension on the anastomosis [22]. The optimal conditions for bile duct reconstruction of BTI are absence of local inflammation and the presence of proximal bile duct dilatation with the possibility of anastomosis on a healthy biliary mucosal.

## 4. Conclusion

The debate continues, but the mentioned studies support the routinary use of IOC for early detection and correction of LC-induced BTI. It may be especially important to use it during the surgeon's learning curve when the risk is known to be the greatest. A meticulous operative technique in the observance of strict guidelines is very important in preventing BTI. The most important principle is the adequate exposure of the operative field. This requires that the porta hepatis was put under tension by both manual liver retraction and passive retraction by the reverse Trendelenburg position. Optimal visualization of the portal structures is also essential. A frontal view of the porta hepatis should be achieved with the liberal use of a 30° angle laparoscope. Exposure of the triangle of Calot is critical for proper identification of the vital structures. Experts have underlined the importance of a lateral traction on the gallbladder infundibulum in order to open Calot's triangle. This places the cystic duct at a right angle to the common bile duct, thereby reducing the occurrence of misidentification. Clear visualization of both the cystic duct and the choledochos should be obtained during clip placement and transection of the cystic duct. Overuse of electrocautery must be avoided during the dissection of Calot's triangle. IOC should be performed after complete dissection of all ductal structures in the triangle of Calot and before any division, and, finally, the dissection should be carried out close to the gallbladder during its removal from the liver bed. Another important principle is a low threshold for conversion to OC, especially when the anatomy remains unclear during the surgical dissection. Conversion to laparotomy, in complicated cases involving inflammatory changes, aberrant anatomy, or excessive bleeding, is not considered as a failure but rather as good surgical judgement in order to ensure the patient's safety. 

In case of intraoperative suspicion of BTI, a cholangiography must be immediately performed to identify the lesion. The recommended surgical strategy depends greatly on the expertise of the surgeon. While all surgeons agree that injuries recognized during the primary intervention have to be immediately treated, there is no consensus on the management of injuries recognized only after the end of the primary intervention. Endoscopic stenting or early surgical repair in the post-laparoscopic inflammatory stage have been often performed even if it is well established that delayed primary repair gives the best results. In the case of a delayed diagnosis, preoperative imaging is obtained by percutaneous transhepatic cholangiography and endoscopic retrograde cholangio-pancreatography. A precise diagnosis is crucial, and all intrahepatic ducts must be visualized. The optimal timing to do biliary repair has not been clearly established. However, in certain patients with a more distal BTI and a well-vascularized and noninflammed bile duct, repair without dissection of the hilar plate should be performed. In patients presenting an established stenosis following a previous surgical repair several months earlier, percutaneous dilatation should be indicated. Finally, percutaneous dilatation of long-term stenosiss (ductal-to-ductal or hepato-enteric) may also be effective.

## Figures and Tables

**Table 1:** Incidence of BTI during OC.

Authors	Year	Country	Number of OCs	Patients with BTI (%)
Rosenquist and Myrin [2]	1960	Sweden	21530	43 (0.20%)
Bismuth [3]	1981	France	53637	84 (0.16%)
Sandberg [4]	1985	Sweden	92856	65 (0.07%)
Clavien [5]	1992	USA/Switzerland	1088	0 (0%)
Roslyn [6]	1993	USA	42474	91 (0.2%)
Gouma [7]	1994	The Netherlands	8780	45 (0.5%)
